# Community-based diabetes self-management and support program: addressing quality of life and social vulnerability

**DOI:** 10.1186/s12889-025-22627-1

**Published:** 2025-04-25

**Authors:** Samantha Kanny, Luke Hall, Dawn Blackhurst, Windsor Westbrook Sherrill

**Affiliations:** 1 Department of Public Health Sciences, Clemson University, Clemson, SC USA; 2 Prisma Health, Greenville, SC USA; 3 The Dartmouth Institute for Health Policy and Clinical Practice, Lebanon, New Hampshire USA

**Keywords:** Diabetes, Self-management, Community health, Social vulnerability, Quality of life, Diabetes mellitus/prevention and control

## Abstract

**Background:**

In the United States, 38.4 million people have been diagnosed with diabetes, and it continues to rise. The increasing rate of diabetes has become a significant public health challenge due, in part, to the association between diabetes and decreased levels of physical and emotional well-being. Currently, there are few assessments of the impact of diabetes self-management programs on individuals with diabetes quality of life and social vulnerability. This study examined pre- to post-program quality of life outcomes for participants in a community-based diabetes-self management and support (DSMS) program and assessed the association between the change in quality of life pre- to post-program and social vulnerability.

**Methods:**

Health Extension for Diabetes (HED) is a 4-month, community-based DSMS program delivered in the Southeast region of the United States. HED includes standardized education and personalized support to help participants manage their diabetes. The 12-Item Short Form Health Survey (SF-12) was utilized to assess participants’ physical and mental quality of life pre- and post-program participation. The Centers for Disease Control and Prevention’s (CDC) Social Vulnerability Index (SVI) was used to determine individuals’ social vulnerability level (low: 0–0.25, low-to-moderate: 0.2501–0.5, moderate-to-high: 0.501–0.75, high: 0.7501–1.0). Wilcoxon sign-ranked tests assessed changes in SF-12 pre- and post-HED and linear regressions examined the association between quality of life and social vulnerability level.

**Results:**

SF-12 scores indicated significant positive changes in physical and mental quality of life for all program participants (*N* = 1,006). All SVI subgroups were observed to have significant improvements in physical health scores. Individuals with moderate-to-high and high SVI scores showed significant improvement in mental health scores, while individuals with low and low-to-moderate SVI scores did not.

**Conclusion:**

Participants of the community-based diabetes self-management and support program experienced improvements in quality of life across varying levels of social vulnerability, as measured by the SVI. While integrating upstream social determinants of health considerations into DSMS program design and delivery addresses health disparities, future research should consider the implementation of more general mental health resources to address the psychological burden associated with living with chronic disease.

## Background

In the past 25 years, all forms of diabetes have increased in prevalence, making the chronic condition a significant public health challenge in the United States and globally [1, 2]. As of 2021, global diabetes prevalence was approximately 10.5% (536.6 million people), and 11.6% of individuals living in the United States (38.4 million people) were living with diabetes [3, 4]. Literature has shown that areas with a high level of social vulnerability, which refers to “the potential negative effects on communities caused by external stresses on human health” [5](p803), are strongly associated with diabetes prevalence [6]. Social vulnerability has increasingly become an essential consideration in guiding public health interventions to target and support vulnerable populations [7, 8]. Evidence suggests that higher social vulnerability is related to adverse effects for those living with diabetes, such as increased symptom burden and attrition rates in diabetes management programs [9, 10]. As diabetes continues to become more prevalent among populations with health disparities, interventions that address social vulnerabilities are increasingly important.

A composite measurement created by the Centers for Disease Control and Prevention (CDC) called the Social Vulnerability Index (SVI) has been validated to measure social vulnerability through individual social variables including community resilience, socioeconomic, and demographic factors [11]. Social vulnerability has been purported to sufficiently represent the overall impact of multiple social determinants of health at a community level [11]. Research has shown that challenges related to social vulnerability can exacerbate diabetes and its complications [9], yet there remains a nascent knowledge gap on the possible effects of social vulnerability on successful participation in diabetes management programs. Thus, understanding the potential relationship between each individual’s social vulnerability and success in diabetes management programs is critical to providing the proper resources and support for participants in such programs to manage their diabetes effectively.

One type of program created to help individuals overcome the difficulties in diabetes self-management is Diabetes Self-Management and Support (DSMS) [12]. Recommended by the American Diabetes Association (ADA), DSMS provides individuals with the foundation for managing their diabetes and navigating the decisions and activities that come with chronic disease [13]. DSMS exists to help an individual “… implement informed decision making, self-management behaviors, problem-solving, and active collaboration with the health care team to improve clinical outcomes, health status, and quality of life.” ^14(p1639)^ DSMS programs can offer support in various ways including behavioral, educational, psychosocial, or clinical support [15]. Through these programs, participants are expected to experience improvements in diabetes knowledge, coping skills, and diabetes self-management [13, 14]. Research has shown that improvement in these areas can lead to an overall improvement in individuals’ physical and emotional well-being [16]. DSMS, psychosocial care, health coaching, and the collaborative development of health behavior goals and care plans are expert-recommended standards of care endorsed by the ADA [17]. 

Previous studies have found that those living with diabetes often report low levels of both physical and emotional well-being [18, 19]. A standard instrument to examine physical and mental quality of life (QOL) is the 12-item Short Form Health Survey (SF-12) [20]. Developed in 1996, SF-12 is a validated tool for measuring quality of life in various populations with chronic conditions [21]. Additionally, SF-12 can be employed to measure the relationship between physical and mental health function and social determinants of health [22]. A study by Markle-Reid et al. (2017) utilized SF-12 to examine the effect of a 6-month community-based intervention for individuals with Type 2 diabetes and their quality of life [23]. The study observed that participation in the six-month community-based program improved participant quality of life and reduced symptoms of depression in older adults with diabetes. However, no significant improvement in physical health was found [23]. Similarly, one community-based diabetes education program found a modest positive impact on mental health in participants post-program [24]. Although some studies have examined associations of QOL in those living with diabetes and individual-level or psychosocial factors, a recent systematic review affirms the challenge of measuring the full breadth of QOL and mental health outcomes for this population [25–29]. Lastly, a diabetes medication therapy management program found significant improvement in the mental health component of the SF-12 but no significant improvement in the physical health dimension of their participants [30]. Although these studies examined various health disparities as potential covariates, they did not explore the use of the SF-12 in investigating the potential association between physical and mental health functioning and social vulnerability.

Evidence suggests that particular components of social determinants of health are significantly associated with diabetes management and outcomes; however, there is growing interest in the broader potential associations between social vulnerability, social determinants of health, and the impact of diabetes interventions and outcomes [31–33].

## Methods

### Study aim and design

This study aimed to evaluate quality of life outcomes pre- to post-program for participants in a community-based DSMS program. The association between the change in quality of life pre- to post-program and social vulnerability level was also examined. This work is part of a larger longitudinal, mixed-methods evaluation study of the Health Extension for Diabetes (HED) program.

### Intervention

HED is a 4-month, community-based DSMS program comprised of eight bi-weekly core group educational sessions with extensive participant interaction through individual, personalized follow-up support between group sessions. HED is designed to be a high-touch, adaptable program providing continuous support beyond structured sessions, ensuring participants receive ongoing guidance tailored to their needs.

The program was created with eight standardized educational sessions and includes robust pre- and post-program participant data evaluation. Data collection occurs at program registration, program graduation, 6-months post-program, and 1-year post-program by HED facilitators and HED student interns. Data for the present study was collected at registration and graduation from the program.

The curriculum was constructed based on the Association of Diabetes Cares and Education Specialists’ (ADCES) Seven Self-Care Behaviors for Managing Diabetes, which include healthy coping, healthy eating, being active, taking medication, monitoring, problem-solving, and reducing risks [34]. HED has been recognized by the American Diabetes Association as a Practice-Tested Support Program for individuals with Type 1 and Type 2 diabetes [35]. It was created in collaboration between Clemson University, a state land-grant university, and a regional healthcare system. A key strength of HED is its close integration with clinical partners, ensuring that at least one session is delivered by a Certified Diabetes Care and Education Specialist (CDCES), who are called clinical diabetes educators within the HED program. These clinical diabetes educators are also available through the program to answer individualized clinical questions and are certified to work with both Type 1 and Type 2, allowing the program to support both types of diabetes. This collaboration enhances participants’ access to expert guidance on diabetes management.

HED is facilitated by community health workers who hold a bachelor’s degree and have been trained in diabetes prevention and self-care behaviors. In HED, community health workers are referred to as facilitators. These facilitators are recruited from local communities, ensuring cultural competence and relatability. They undergo extensive training, including HED Facilitator Training with a master trainer (i.e. a facilitator who has independently conducted 3 cohorts and received approval to train new facilitators), shadowing at least one full cohort before independently leading a program, and completing subject-matter training aligned with the ADCES7 Self-Care Behaviors and ADCES Prevention 101: Fundamentals of Diabetes and Prediabetes.

### Study setting and characteristics of participants

HED participants represented 37 of the 46 counties in South Carolina. Data used for this study is part of an ongoing longitudinal evaluation study initiated in October 2017. HED is delivered in partnership with and at community sites such as activity centers, churches, and other local organizations. Recruitment for the program occurred at these locations, as well as at health fairs across the state. Eligibility for program participation required individuals to be at least 18 years old and have a clinical or self-reported diagnosis of type 1 or type 2 diabetes mellitus. Individuals who were institutionalized (i.e. in a psychiatric facility, were incarcerated, or reported long-term hospitalization), women who were pregnant, and individuals with end-stage renal disease were excluded. This study was conducted in accordance with institutional guidelines for human subject protection and was approved by the Prisma Health Institutional Review Board (IRB) (Approval No: 1852786-8). All participants gave informed consent prior to participation in this study.

### Description of measures

#### Demographic information

Baseline demographic data was obtained from all participants at registration for HED by program facilitators and HED student interns. Demographic information was self-reported and included age, weight, BMI, biological sex, race, ethnicity, educational attainment, annual income, zip code, family history of diabetes, history of gestational diabetes, and history of hypertension. Social Vulnerability Index scores were assigned to each participant based on their self-reported zip code.

#### SF-12

The SF-12 is a health-related QOL measure. The 12-item instrument consists of questions that measure eight health domains to assess physical and mental health. SF-12 yields two scores: (1) the Physical Component Summary (PCS), which assesses physical health, and (2) the Mental Component Summary (PCS), which assesses mental health. PCS score domains include General Health, Physical Functioning, Role Physical, and Body Pain. In contrast, the MCS score domains include Vitality, Social Functioning, Role Emotional, and Mental Health [36]. The instrument has been validated across several chronic diseases and conditions, including diabetes [37, 38]. SF-12 scores have been observed to have good internal consistency (α: PCS = 0.85; MCS = 0.83) and acceptable test-retest reliability (intraclass correlation coefficient (ICC): PCS = 0.72; MCS = 0.63) in individuals with self-reported diabetes [39]. Scoring was completed by the research team using the SF-12 scoring algorithm, Optum’s QualityMetric [40]. A norm-based scoring system is used to interpret SF-12 scores (mean: 50; range: 0–100). Scores above 50 indicate better-than-average health and scores below 50 indicate below-average health [41]. Participants in this study completed the SF-12 instrument pre-program (i.e. at registration for HED) and post-program (i.e. at graduation from HED).

#### CDC’s social vulnerability index (SVI)

The CDC’s Social Vulnerability Index was employed as a composite measure for social vulnerability. The SVI has been validated to measure individual social variables, such as community resilience, and socioeconomic, and demographic factors that yield an overall vulnerability score [11]. More specifically, the composite considers pre-existing granular geocoded data in the following four themes: (1) socioeconomic status, (2) household composition and disability, (3) minority status and language, and (4) mobility and transportation [42]. The psychometrics of the SVI have been evaluated, and it was observed to perform favorability in measures of validity [43]. The CDC has assigned each United States census tract an SVI score from 0 (lowest vulnerability) to 1 (highest vulnerability). Table 1 describes the four social vulnerability levels related to SVI and their associated SVI score [44]. Research has shown that social vulnerability measures, like the SVI, may have differential effects on evidence-based diabetes prevention and management programs [45]. 

**Table 1:** SVI categories and associated scores

CDC SVI County Score (Range: 0–1)	Category Name
0.00–0.25	Low Social Vulnerability
0.2501–0.50	Low-to-Moderate Social Vulnerability
0.501–0.75	Moderate-to-High Social Vulnerability
0.7501–1.00	High Social Vulnerability

### Statistical analysis

Descriptive statistics were computed for continuous and categorical demographic variables. Distribution of SF-12 PCS and MCS scores were evaluated using Histograms, Q-Q Plots, and Shapiro-Wilk tests and determined to deviate from a normal distribution. Wilcoxon sign-ranked tests were used to assess pre-post differences in SF-12 domains: the physical health component (PCS) and mental health component (MCS). An a priori analysis indicated a total sample size of 88 participants was needed to adequately power the Wilcoxon sign-ranked tests (Cohen’s $\:{f}^{2}$ = 0.4). Cohen’s d was calculated to determine the effect size of the mean change in QOL outcomes pre- to post-program (0.2 = small; 0.4 = medium; ≥ 0.8 = large) [46]. Participants’ SVI scores were categorized into the four SVI levels assigned by the CDC: low, low-to-moderate, moderate-to-high, and high (low: 0–0.25, low-to-moderate: 0.2501–0.5, moderate-to-high: 0.501–0.75, high: 0.7501–1.0).

Linear regressions were conducted to examine the extent to which social vulnerability (SVI category) predicted the change in PCS and MCS scores pre- to post-program. An a priori analysis indicated a total sample size of 35 participants was needed to power the linear regressions adequately (Cohen’s $\:{f}^{2}$ = 0.4). Statistical significance was set at *P* < 0.05. A priori analyses were conducted using G*Power [47]. All other analyses were conducted using SAS software, Version 9.4 of the SAS System for Windows (SAS Institute, Cary, NC, USA).

## Results

Of the 1,581 participants who completed HED registration data collection and enrolled in the program, 399 (25%) participants dropped out of HED, 153 (10%) participants were actively participating in HED, 12 (1%) participants were missing SF-12 registration data, and 11 (1%) participants were missing zip codes so their SVI scores could not be calculated and were excluded from the analysis. A total of 1,006 (63%) participants completed both pre- and post-program SF-12 surveys and were included in this study for analysis.

A majority (72%) of participants were female. Over half (56%) identified as White, and 34% identified as Black or African American. Most participants (89%) identified as not Hispanic or Latino. Approximately 7% had less than a high school education or some high school education, while almost 18% of the sample had a high school diploma or GED, and 34% had some college or a technical/associate degree. Nearly a quarter (24%) of the population reported an annual income of less than $25,000. A family history of diabetes was present in 78%, and a history of hypertension was reported in 74% of the population. Of females, about 20% reported a history of gestational diabetes. Table 2 provides a comprehensive overview of the baseline demographic characteristics of participants in the study.

**Table 2:** Characteristics of individuals enrolled in HED

HED Individual Characteristics	All Participants *N* = 1,*006*
Age, Mean (Std.)	64.69 (± 12.34)
Weight, Mean (Std.)	210 (± 51.21)
BMI, Mean (Std.)	34.30 (± 8.16)
Sex, N (%)	
Males	271 (26.9)
Females	723 (71.9)
Prefer Not to Answer	0 (0.0)
Missing	12 (1.2)
Race, N (%)	
White	567 (56.4)
Black or African-American	344 (34.2)
Other	73 (7.3)
Prefer Not to Answer	11 (1.1)
Missing	11 (1.1)
Ethnicity, N (%)	
Hispanic or Latino	71 (7.1)
Not Hispanic or Latino	890 (88.5)
Other	16 (1.6)
Prefer Not to Answer	18 (1.8)
Missing	11 (1.1)
Educational Attainment, N (%)	
Less than High School/Some High School	74 (7.4)
High School Diploma/GED	189 (18.8)
Some College	196 (19.5)
Technical Degree/Associate Degree	145 (14.4)
Bachelor’s Degree	192 (19.1)
Some Postgraduate Education	199 (19.8)
Prefer Not to Answer	0 (0.0)
Missing	11 (1.1)
Annual Income, N (%)	
< $25, 000	238 (23.7)
$25,000 - $50,000	187 (18.6)
> $50,000	239 (23.8)
Prefer Not to Answer	331 (32.9)
Missing	11 (1.1)
Family History of Diabetes, N (%)	
No	209 (20.8)
Yes	785 (78.0)
Prefer Not to Answer	0 (0.0)
Missing	12 (1.2)
History of Gestational Diabetes, N (%)	
No	576 (79.7)
Yes	147 (20.3)
Prefer Not to Answer	0 (0.0)
Missing	0 (0.0)
History of Hypertension, N (%)	
No	254 (25.2)
Yes	740 (73.6)
Prefer Not to Answer	0 (0.0)
Missing	12 (1.2)

### Pre-/post-intervention changes

**Fig. 1:**
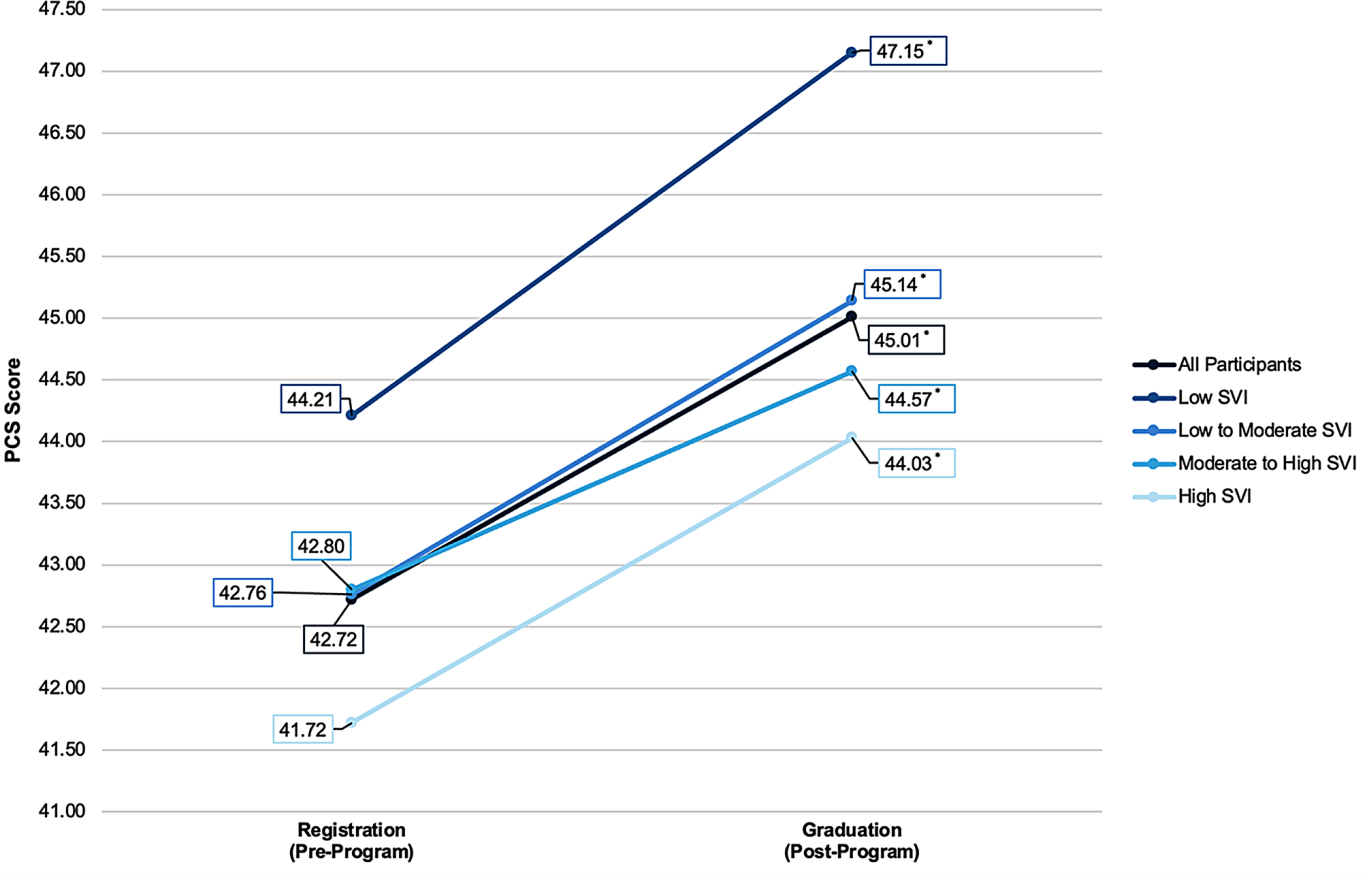
Average physical component scores pre to post-HED program. Mean SF-12 Physical Component scores at pre-program (registration) and post-program (graduation) for all participants, as well as stratified by levels of social vulnerability measured by the Social Vulnerability Index. Asterisks (*) denote significant change in PCS scores from pre- to post-program (*P* < 0.05). *Abbreviations*: PCS, Physical Component scores

**Fig. 2:**
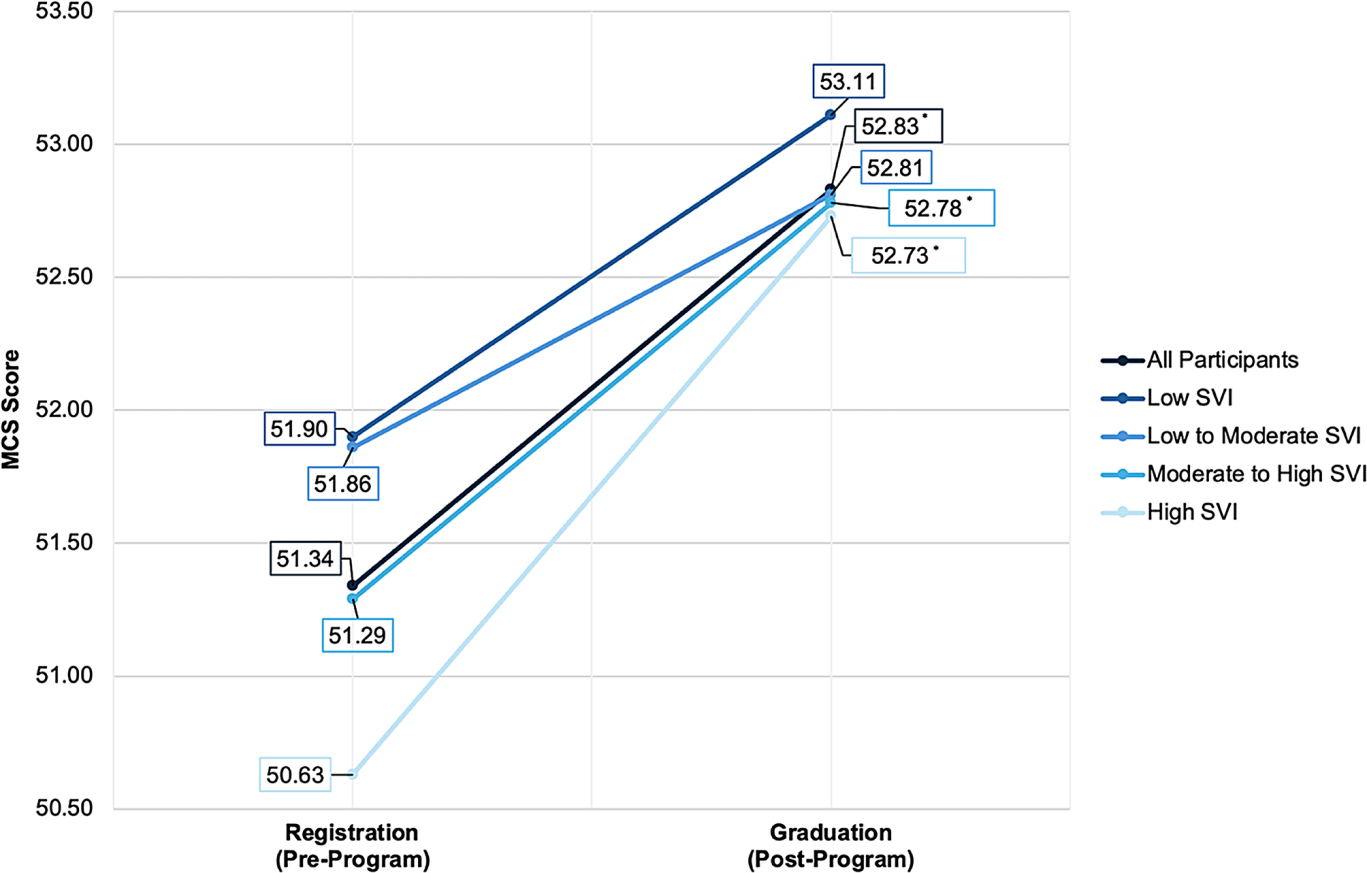
Average Mental Component scores pre to post-HED program. Mean SF-12 Mental Component scores at pre-program (registration) and post-program (graduation) for all participants, as well as stratified by levels of social vulnerability measured by the Social Vulnerability Index. Asterisks (*) denote significant change in MCS scores from pre- to post-program (*P* < 0.05). *Abbreviations*: MCS, Mental Component scores

A Wilcoxon signed-rank test indicated significant changes in quality of life outcomes for physical health (Z = -8.84, *P* < 0.001) and mental health (Z = -4.86, *P* < 0.001) for all individuals (Table 3). The average physical and mental health scores significantly increased pre- to post-HED for all individuals (2.29 and 1.49, respectively). Figure 1 displays the average Physical Component scores pre- to post-HED program. Figure 2 displays the average Mental Component scores pre- to post-HED program.

**Table 3:** Pre-post health extension for diabetes averages of SF-12 physical and mental component scores

Characteristics	Pre-HED Mean (Std.)	Post-HED Mean (Std.)	Mean Change Mean (Std.)	Z-value	*P*-value	Effect Size Cohen’s d
***All Participants*** (***N*** ***= 1***,***006)***						
PCS	42.72 (± 11.02)	45.01 (± 11.01)	2.29 (± 8.52)	-8.84	**< 0.001**	0.269
MCS	51.34 (± 10.40)	52.83 (± 9.40)	1.49 (± 9.04)	-4.86	**< 0.001**	0.165
**Social Vulnerability Index**						
***Low SVI*** (***N*** ***= 183)***						
PCS	44.21 (± 10.96)	47.15 (± 10.62)	2.94 (± 8.59)	-5.34	**< 0.001**	0.342
MCS	51.90 (± 10.00)	53.11 (± 8.88)	1.21 (± 9.68)	-1.21	0.227	0.125
***Low-to-Moderate SVI*** (***N*** ***= 242)***						
PCS	42.76 (± 10.53)	45.14 (± 10.80)	2.37 (± 8.44)	-4.36	**< 0.001**	0.281
MCS	51.86 (± 9.89)	52.81 (± 9.03)	0.95 (± 9.53)	-1.51	0.131	0.100
***Moderate-to-High SVI*** (***N*** ***= 282)***						
PCS	42.80 (± 11.11)	44.57 (± 11.17)	1.77 (± 8.36)	-3.74	**< 0.001**	0.211
MCS	51.29 (± 10.64)	52.78 (± 10.65)	1.49 (± 8.01)	-2.74	**0.006**	0.186
***High SVI*** (***N*** ***= 299)***						
PCS	41.72 (± 11.31)	44.03 (± 11.12)	2.32 (± 8.72)	-4.379	**< 0.001**	0.266
MCS	50.63 (± 10.80)	52.73 (± 9.63)	2.10 (± 9.16)	-3.901	**< 0.001**	0.229

*Abbreviations* PCS, Physical Component score; MCS, Mental Component score; SVI, Social Vulnerability Index

Among SVI subscales, no significant improvement in mental health scores was indicated for individuals in the low and low-to-moderate social vulnerability levels. However, significantly higher post-program MCS scores were observed in individuals in the moderate-to-high social vulnerability level (Z = -2.74, *P* = 0.006) and high social vulnerability level (Z = -3.901, *P* < 0.001) compared to pre-program MCS scores. The average mental health score increased by 1.49 points for individuals in the moderate-to-high social vulnerability level and 2.10 points for individuals in the high social vulnerability level. In contrast, all SVI groups had significant improvements in physical health scores (*P values* < 0.05).

### Linear regressions

Results for linear regression analyses can be found in Table 4. The linear regression analysis indicated no significant relationship between social vulnerability level and change in PCS scores, F(1, 1004) = 0.734, *P* = 0.392. Similarly, the regression analysis also revealed no significant relationship between social vulnerability level and change in MCS scores, F(1, 1004) = 1.792, *P* = 0.181.

**Table 4:** Linear regressions examining the association between mean change in SF-12 PCS, MCS and SVI categories

Predictor	B	SE B	β	t-value	*p*-value	95% CI		R^2^
						Lower Bound	Upper Bound	
**PCS**								
SVI	-0.213	0.248	-0.027	-0.857	0.392	-0.700	0.275	0.027
**MCS**								
SVI	0.353	0.263	0.042	1.339	0.181	-0.164	0.870	0.042

*Abbreviations *CI, Confidence Interval; PCS, Physical Component score; MCS, Mental Component score; SVI, Social Vulnerability Index

## Discussion

This study aimed to examine the associations of a community-based diabetes support program on QOL outcomes, considering specific participant social determinants of health as operationalized by the SVI. Poor quality of life can impair individuals’ ability to manage their diabetes effectively and, therefore, must be considered in the design, delivery, and evaluation of any diabetes intervention. Overall, the HED program significantly improved participants’ physical and mental quality of life as measured by SF-12. On average, individuals participating in the program reported a small but significant increase of 2.29 points in their physical health score and a 1.49-point increase in their mental health score. Our findings corroborate a systematic review by Allison et al. (2024), which indicated that diabetes self-management programs for older adults, similar to HED, demonstrate small but meaningful reductions in self-reported outcomes [48]. Conversely, these results differed slightly from previous literature, which observed that community-based diabetes programs significantly improved mental health scores but did not significantly improve physical health scores [23, 24]. Our findings showed an increase in physical health scores for all individuals and across all SVI levels.

Interestingly, individuals in the moderate-to-high and high social vulnerability groups experienced a significant improvement in their mental health scores, while individuals in the low and low-to-moderate social vulnerability groups did not. SVI scores consider community factors such as poverty, lack of access to healthcare, lower education levels, minority status, and transportation/housing insecurity [49]. Thus, individuals from these communities may be more at risk of social vulnerability. HED facilitators aim to empower individuals in the program to manage their diabetes through education, providing information about available resources (i.e. free healthcare clinics, food banks, exercise programs, and publicly accessible transportation), and improving their self-efficacy to manage their diabetes [50]. Thus, these results may indicate that HED was effective in relieving the burden of modifiable social vulnerabilities such as access to healthcare, food resources, and diabetes education. However, our findings suggest that HED does not address mental health aspects beyond those caused by community-level social determinants of health.

While results from this study indicate HED was successful in improving physical health across all social vulnerability levels, adherence to physical activity routines remains particularly challenging for those within high social vulnerability communities. André Luiz Galvim and colleagues highlighted such obstacles including inadequate spaces for exercise, a lack of awareness of the benefits of physical activity, the complexities of time management, and high obesity rates in these populations [51]. HED facilitators connect participants to a wide variety of free exercise programs, teach simple, low-stress exercises that can be completed at home, and educate their participants on the benefits of exercise on their diabetes. Findings from this study suggest that offering a wide variety of exercise options, in conjunction with increasing participants’ knowledge of the importance of exercises, may enable individuals—independent of their social vulnerability level—to select an exercise regime that works best for them and improve adherence to participation in physical activity. The potential ability to overcome the barriers associated with adherence to participation in physical activity underscores the necessity for interventions tailored to the distinctive needs of each individual to bolster physical health outcomes.

Our study highlights the differential impact of a community-based diabetes support program on mental health outcomes across varying degrees of social vulnerability. Despite the overall improvement in quality of life, individuals from lower social vulnerability communities did not exhibit significant enhancements in mental health scores. This may stem from resources provided by HED, which are aimed at alleviating the challenges seen with health disparities (e.g. food insecurity and limited access to healthcare). Access to resources and training on how to navigate local services improves mental health by reducing the stress of managing a chronic disease [52]. Thus, our findings suggest that the coping strategies currently offered by HED might not sufficiently enhance mental well-being among individuals who already have access to essential resources and do not face the same hurdles as those with high social vulnerability. Well-being interventions can be broadly applicable to a range of psychological distress and are designed to promote positive mental health such as optimism, gratitude, and resilience. Previous literature observed that well-being interventions, despite their effectiveness in improving outcomes in individuals with diabetes, do not include behavioral strategies that target diabetes self-management [53]. The findings from this study advocate for adaptive strategies in DSMS programs to address the different psychological barriers seen across social vulnerability levels, potentially involving adding more mental health components such as well-being activities.

This study has several strengths. The robustness of findings is due, in part, to the large sample size. Additionally, the integration of both clinical and community-based components allows for a real-world perspective on diabetes self-management support. However, several limitations must be acknowledged. The study population is geographically and demographically homogenous, which may restrict generalizability to more diverse populations. Another limitation is the potential for selection bias, as only participants who attended most scheduled sessions were included in the analysis, potentially overrepresenting individuals with higher motivation or access to healthcare resources. Future research should explore the long-term impact of DSMS programs, incorporating more diverse populations, and examining differential effects between individuals who graduate and those who drop out of DSMS programs to further refine diabetes self-management interventions.

## Conclusion

The present study demonstrates that a community-based DSMS program can significantly improve the overall quality of life for its participants despite their social vulnerability. Health Extension for Diabetes has proven to be an effective DSMS program that enhances program participants’ physical and mental quality of life. This study revealed variations in physical and mental quality-of-life outcomes among participants across social vulnerability levels. Results point to the potential utility of an index such as the SVI as a preparatory tool to inform program recruitment, participation, and support. This proactive approach could help identify individuals at greater risk and enable tailored support, contributing to more targeted efforts to enhance health equity.

## Data Availability

The datasets generated and/or analyzed during the current study are not publicly available due to the restrictions associated with participant consent but are available from the corresponding author on reasonable request.

## Abbreviations

HED: Health Extension for Diabetes
SVI: Social Vulnerability Index
DSMS: Diabetes Self-Management and Support
SF-12: 12-item Short Form Health Survey
DSMES: Diabetes Self-Management Education and Support
ADA: American Diabetes Association
SF-12: 12-Item Short Form Health Survey
QOL: Quality of Life
PCS: SF-12 Physical Health Component
MCS: SF-12 Mental Health Component

## References

[1] Zimmet P, Alberti KG, Shaw J. Global and societal implications of the diabetes epidemic. Nature. 2001;414(6865):782–7. 10.1038/414782a. 11742409 10.1038/414782a

[2] Tomic D, Shaw JE, Magliano DJ. The burden and risks of emerging complications of diabetes mellitus. Nat Rev Endocrinol. 2022;18(9):525–39. 10.1038/s41574-022-00690-7. 35668219 10.1038/s41574-022-00690-7 PMC9169030

[3] Centers for Disease Control and Prevention. National diabetes statistics report. U.S. Department of Health and Human Services; 2024. https://www.cdc.gov/diabetes/data/statistics-report/index.html.

[4] Sun H, Saeedi P, Karuranga S, et al. IDF diabetes atlas: global, regional and country-level diabetes prevalence estimates for 2021 and projections for 2045. Diabetes Res Clin Pract. 2022;183:109119. 10.1016/j.diabres.2021.109119. 34879977 10.1016/j.diabres.2021.109119 PMC11057359

[5] Wolkin A, Collier S, House JS, et al. Comparison of National vulnerability indices used by the centers for disease control and prevention for the COVID-19 response. Public Health Rep. 2022;137(4):803–12. 10.1177/00333549221090262. 35514159 10.1177/00333549221090262 PMC9257512

[6] Tonaco LAB, Vieira MAS, Gomes CS, et al. Social vulnerability associated with the self-reported diagnosis of type II diabetes: a multilevel analysis. Rev Bras Epidemiol. 2021;24:e210010. 10.1590/1980-549720210010.supl.1. 33886883 10.1590/1980-549720210010.supl.1

[7] Mah J, Rockwood K, Stevens S, Keefe J, Andrew MK. Do interventions reducing social vulnerability improve health in community dwelling older adults?? A systematic review. Clin Interv Aging. 2022;17:447–65. 10.2147/CIA.S349836. 35431543 10.2147/CIA.S349836 PMC9012306

[8] Haggerty J, Minotti SC, Bouharaoui F. Development of an individual index of social vulnerability that predicts negative healthcare events: a proposed tool to address healthcare equity in primary care research and practice. Int J Equity Health. 2023;22(1):157. 10.1186/s12939-023-01965-9. 37596614 10.1186/s12939-023-01965-9 PMC10436429

[9] Lenzi FR, Filardi T. Social determinants of vulnerabilities in type 2 diabetes: a call to action. J Endocrinol Invest. 2023;46(4):841–4. 10.1007/s40618-022-01952-x. 36318450 10.1007/s40618-022-01952-x

[10] Flaus-Furmaniuk A, Fianu A, Lenclume V, et al. Attrition and social vulnerability during 2-year-long structured care in type 2 diabetes, the ERMIES randomized controlled trial. BMC Endocr Disord. 2022;22(1):314. 10.1186/s12902-022-01211-3. 36510180 10.1186/s12902-022-01211-3 PMC9746115

[11] Flanagan BE, Hallisey EJ, Adams E, Lavery A. Measuring community vulnerability to natural and anthropogenic hazards: the centers for disease control and prevention’s social vulnerability index. J Environ Health. 2018;80(10):34–6. 32327766 PMC7179070

[12] Beck J, Greenwood DA, Blanton L, et al. 2017 National standards for diabetes Self-Management education and support. Diabetes Educ. 2017;43(5):449–64. 10.1177/0145721717722968. 28753378 10.1177/0145721717722968

[13] Brunisholz KD, Briot P, Hamilton S, et al. Diabetes self-management education improves quality of care and clinical outcomes determined by a diabetes bundle measure. J Multidiscip Healthc. 2014;7:533–42. 10.2147/JMDH.S69000. 25473293 10.2147/JMDH.S69000 PMC4247143

[14] Powers MA, Bardsley JK, Cypress M, et al. Diabetes Self-management education and support in adults with type 2 diabetes: A consensus report of the American diabetes association, the association of diabetes care & education specialists, the academy of nutrition and dietetics, the American academy of family physicians, the American academy of PAs, the American association of nurse practitioners, and the American pharmacists association. J Am Pharmacists Association. 2020;60(6):e1–18. 10.1016/j.japh.2020.04.018. 10.1016/j.japh.2020.04.018 32527704

[15] Funnell MM, Brown TL, Childs BP, et al. National standards for diabetes Self-Management education. Diabetes Care. 2010;33(Suppl 1):S89–96. 10.2337/dc10-S089. 20042780 10.2337/dc10-S089 PMC2797385

[16] Kueh YC, Morris T, Borkoles E, Shee H. Modelling of diabetes knowledge, attitudes, self-management, and quality of life: a cross-sectional study with an Australian sample. Health Qual Life Outcomes. 2015;13(1):129. 10.1186/s12955-015-0303-8. 26286395 10.1186/s12955-015-0303-8 PMC4543474

[17] American Diabetes Association Professional Practice Committee. 5. Facilitating positive health behaviors and Well-being to improve health outcomes: standards of care in Diabetes-2025. Diabetes Care. 2025;48(Supplement1):S86–127. 10.2337/dc25-S005. 39651983 10.2337/dc25-S005 PMC11635047

[18] Rubin RR, Peyrot M. Quality of life and diabetes. Diabetes Metab Res Rev. 1999;15(3):205–18. 10.1002/(sici)1520-7560(199905/06)15:3%3C205::aid-dmrr29%3E3.0.co;2-o. 10441043 10.1002/(sici)1520-7560(199905/06)15:3<205::aid-dmrr29>3.0.co;2-o

[19] Schram MT, Baan CA, Pouwer F. Depression and quality of life in patients with diabetes: A systematic review from the European depression in diabetes (EDID) research consortium. Curr Diabetes Rev. 2009;5(2):112–9. 10.2174/157339909788166828. 19442096 10.2174/157339909788166828 PMC2764861

[20] Farivar SS, Cunningham WE, Hays RD. Correlated physical and mental health summary scores for the SF-36 and SF-12 health survey, V.1. Health Qual Life Outcomes. 2007;5(1):54. 10.1186/1477-7525-5-54. 17825096 10.1186/1477-7525-5-54 PMC2065865

[21] Turner-Bowker D, Hogue SJ. Short form 12 health survey (SF-12). In: Michalos AC, editor. Encyclopedia of quality of life and Well-Being research. Springer Netherlands; 2014. pp. 5954–7. 10.1007/978-94-007-0753-5_2698.

[22] Burdine JN, Felix MR, Abel AL, Wiltraut CJ, Musselman YJ. The SF-12 as a population health measure: an exploratory examination of potential for application. Health Serv Res. 2000;35(4):885–904. 11055454 PMC1089158

[23] Markle-Reid M, Ploeg J, Fraser KD, et al. Community program improves quality of life and Self-Management in older adults with diabetes mellitus and comorbidity. J Am Geriatr Soc. 2018;66(2):263–73. 10.1111/jgs.15173. 29178317 10.1111/jgs.15173 PMC5836873

[24] Sugiyama T, Steers WN, Wenger NS, Duru OK, Mangione CM. Effect of a community-based diabetes self-management empowerment program on mental health-related quality of life: a causal mediation analysis from a randomized controlled trial. BMC Health Serv Res. 2015;15(1):115. 10.1186/s12913-015-0779-2. 25880234 10.1186/s12913-015-0779-2 PMC4375843

[25] Alzahrani O, Fletcher JP, Hitos K. Quality of life and mental health measurements among patients with type 2 diabetes mellitus: a systematic review. Health Qual Life Outcomes. 2023;21(1):27. 10.1186/s12955-023-02111-3. 36949507 10.1186/s12955-023-02111-3 PMC10031182

[26] Kempf K, Martin S. Autonomous exercise game use improves metabolic control and quality of life in type 2 diabetes patients - a randomized controlled trial. BMC Endocr Disorders. 2013;13(1):57. 10.1186/1472-6823-13-57. 10.1186/1472-6823-13-57 PMC3880220 24321337

[27] Reliability. Validity of a diabetes quality-of-life measure for the diabetes control and complications trial (DCCT). The DCCT research group. Diabetes Care. 1988;11(9):725–32. 10.2337/diacare.11.9.725. 10.2337/diacare.11.9.725 3066604

[28] Piette JD, Kerr EA. The impact of comorbid chronic conditions on diabetes care. Diabetes Care. 2006;29(3):725–31. 10.2337/diacare.29.03.06.dc05-2078. 16505540 10.2337/diacare.29.03.06.dc05-2078

[29] Ebrahimi H, Ashrafi Z, Rudsari DM, Parsayekta Z, Haghani H. Effect of Family-Based education on the quality of life of persons with type 2 diabetes: A randomized clinical trial. J Nurs Res. 2018;26(2):97–103. 10.1097/jnr.0000000000000212. 28858971 10.1097/jnr.0000000000000212

[30] Johnson CL, Nicholas A, Divine H, Perrier DG, Blumenschein K, Steinke DT. Outcomes from diabetescare: A pharmacist-provided diabetes management service. J Am Pharmacists Association. 2008;48(6):722–30. 10.1331/JAPhA.2008.07133. 10.1331/JAPhA.2008.07133 19019800

[31] Hill-Briggs F, Adler NE, Berkowitz SA, et al. Social determinants of health and diabetes: A scientific review. Diabetes Care. 2020;44(1):258–79. 10.2337/dci20-0053. 33139407 10.2337/dci20-0053 PMC7783927

[32] Hill-Briggs F, Fitzpatrick SL. Overview of social determinants of health in the development of diabetes. Diabetes Care. 2023;46(9):1590–8. 10.2337/dci23-0001. 37354331 10.2337/dci23-0001

[33] Walker RJ, Gebregziabher M, Martin-Harris B, Egede LE. Independent effects of socioeconomic and psychological social determinants of health on self-care and outcomes in type 2 diabetes. Gen Hosp Psychiatry. 2014;36(6):662–8. 10.1016/j.genhosppsych.2014.06.011. 25103544 10.1016/j.genhosppsych.2014.06.011 PMC4254055

[34] ADCES7 Self-Care Behaviors- The Framework for Optimal Self-Management, Accessed ADCES. November 6, 2023. https://www.diabeteseducator.org/practice/practice-tools/app-resources/the-aade7-self-care-behaviors-the-framework-for-optimal-self-management

[35] Diabetes Support Directory| American Diabetes Association. Accessed November 6. 2023. https://professional.diabetes.org/content-page/diabetes-support-directory

[36] Huo T, Guo Y, Shenkman E, Muller K. Assessing the reliability of the short form 12 (SF-12) health survey in adults with mental health conditions: a report from the wellness incentive and navigation (WIN) study. Health Qual Life Outcomes. 2018;16:34. 10.1186/s12955-018-0858-2. 29439718 10.1186/s12955-018-0858-2 PMC5811954

[37] Salyers MP, Bosworth HB, Swanson JW, Lamb-Pagone J, Osher FC. Reliability and validity of the SF-12 health survey among people with severe mental illness. Med Care. 2000;38(11):1141–50. 10.1097/00005650-200011000-00008. 11078054 10.1097/00005650-200011000-00008

[38] Wan EYF, Choi EPH, Yu EYT, et al. Evaluation of the internal and external responsiveness of short Form-12 health survey version 2 (SF-12v2) in patients with type 2 diabetes mellitus. Qual Life Res. 2018;27(9):2459–69. 10.1007/s11136-018-1908-2. 29948606 10.1007/s11136-018-1908-2

[39] Kathe N, Hayes CJ, Bhandari NR, Payakachat N. Assessment of reliability and validity of SF-12v2 among a diabetic population. Value Health. 2018;21(4):432–40. 10.1016/j.jval.2017.09.007. 29680100 10.1016/j.jval.2017.09.007

[40] Ware JJr, Kosinski M, Turner-Bowker D, Gandek B. How to Score Version 2 of the SF-12v2^®^ Health Survey (With a Supplement Documenting SF-12^®^ Health Survey). QualityMetric Incorporated. 2002. https://www.qualitymetric.com/health-surveys/the-sf-12v2-pro-health-survey/

[41] Ware J, Kosinski M, Keller SD. A 12-Item Short-Form health survey: construction of scales and preliminary tests of reliability and validity. Med Care. 1996;34(3):220–33. 10.1097/00005650-199603000-00003. 8628042 10.1097/00005650-199603000-00003

[42] Tran T, Rousseau MA, Farris DP, Bauer C, Nelson KC, Doan HQ. The social vulnerability index as a risk stratification tool for health disparity research in cancer patients: a scoping review. Cancer Causes Control. 2023;34(5):407–20. 10.1007/s10552-023-01683-1. 37027053 10.1007/s10552-023-01683-1 PMC10080510

[43] Centers for Disease Control and Prevention/Agency for Toxic Substances and Disease Registry. A Validity Assessment of the Centers for Disease Control and Prevention/Agency for Toxic Substances and Disease Registry Social Vulnerability Index (CDC/ATSDR SVI). US department of health and human services, centers for disease control and prevention/agency for toxic substances and disease registry. Published Online May 9, 2024. https://www.atsdr.cdc.gov/placeandhealth/svi/publications/publications_materials.html

[44] CDC/ATSDR Social Vulnerability Index (SVI). January 18. 2024. Accessed January 29, 2024. https://www.atsdr.cdc.gov/placeandhealth/svi/index.html

[45] Mandelbaum J. Associations between social vulnerability and providing Evidence-Based diabetes prevention and management activities in South Carolina, 2019. Prev Chronic Dis. 2023;20. 10.5888/pcd20.220199. 10.5888/pcd20.220199 PMC9983598 36821523

[46] Sullivan GM, Feinn R. Using effect Size—or why the P value is not enough. J Grad Med Educ. 2012;4(3):279–82. 10.4300/JGME-D-12-00156.1. 23997866 10.4300/JGME-D-12-00156.1 PMC3444174

[47] Faul F, Erdfelder E, Lang AG, Buchner A. G*Power 3: A flexible statistical power analysis program for the social, behavioral, and biomedical sciences. Behav Res Methods. 2007;39(2):175–91. 10.3758/BF03193146. 17695343 10.3758/bf03193146

[48] Alliston P, Jovkovic M, Khalid S, Fitzpatrick-Lewis D, Ali MU, Sherifali D. The effects of diabetes self-management programs on clinical and patient reported outcomes in older adults: a systematic review and meta-analysis. Front Clin Diabetes Healthc. 2024;5:1348104. 10.3389/fcdhc.2024.1348104. 38952998 10.3389/fcdhc.2024.1348104 PMC11215190

[49] Flanagan BE, Gregory EW, Hallisey EJ, Heitgerd JL, Lewis B. A Social Vulnerability Index for Disaster Management. *Journal of Homeland Security and Emergency Management*. 2011;8(1). Accessed February 22, 2025. https://stacks.cdc.gov/view/cdc/134506

[50] Funnell MM, Tang TS, Anderson RM. From DSME to DSMS: developing Empowerment-Based diabetes Self-Management support. Diabetes Spectr. 2007;20(4):221–6. 10.2337/diaspect.20.4.221.

[51] Galvim AL, Oliveira IM, Martins TV, et al. Adherence, adhesion, and dropout reasons of a physical activity program in a high social vulnerability context. J Phys Act Health. 2019;16(2):149–56. 10.1123/jpah.2017-0606. 30626259 10.1123/jpah.2017-0606

[52] Kreuter MW, Thompson T, McQueen A, Garg R. Addressing social needs in health care settings: evidence, challenges, and opportunities for public health. Annu Rev Public Health. 2021;42:329–44. 10.1146/annurev-publhealth-090419-102204. 33326298 10.1146/annurev-publhealth-090419-102204 PMC8240195

[53] Massey CN, Feig EH, Duque-Serrano L, Wexler D, Moskowitz JT, Huffman JC. Well-Being interventions for individuals with diabetes: A systematic review. Diabetes Res Clin Pract. 2019;147:118–33. 10.1016/j.diabres.2018.11.014. 30500545 10.1016/j.diabres.2018.11.014 PMC6370485

